# The growing ubiquity of algorithms in society: implications, impacts and innovations

**DOI:** 10.1098/rsta.2017.0364

**Published:** 2018-08-06

**Authors:** S. C. Olhede, P. J. Wolfe

**Affiliations:** 1 Department of Statistical Science, University College London, London, UK; 2 Department of Computer Science, University College London, London, UK; 3 Department of Mathematics, University College London, London, UK; 4 Department of Statistics, Purdue University, West Lafayette, IN, USA; 5 Department of Computer Science, Purdue University, West Lafayette, IN, USA; 6 School of Electrical and Computer Engineering, Purdue University, West Lafayette, IN, USA

**Keywords:** accountability, data science, ethics, governance, privacy, transparency

## Abstract

The growing ubiquity of algorithms in society raises a number of fundamental questions concerning governance of data, transparency of algorithms, legal and ethical frameworks for automated algorithmic decision-making and the societal impacts of algorithmic automation itself. This article, an introduction to the discussion meeting issue of the same title, gives an overview of current challenges and opportunities in these areas, through which accelerated technological progress leads to rapid and often unforeseen practical consequences. These consequences—ranging from the potential benefits to human health to unexpected impacts on civil society—are summarized here, and discussed in depth by other contributors to the discussion meeting issue.

This article is part of a discussion meeting issue ‘The growing ubiquity of algorithms in society: implications, impacts and innovations'.

## 1. Introduction

The growing ubiquity of algorithms in society, treated in this discussion meeting issue [1–13], raises a number of fundamental questions. Data, in the form of observations about our world, permeate modern society. This yields a tremendous potential for making better decisions [14]. However, to make sense of data requires the use of algorithms to extract information from observations. This information can in turn be used to make informed—and in some cases even fully automated—decisions. The theory and practice of such algorithms has given birth to the modern discipline of data science, a blend of statistics, computer science and mathematics.

The large-scale availability of data, coupled with rapid technological advances in algorithms, is changing society markedly [15–19]. The potential benefits of automated decision-making are myriad and clear, and yet at the same time, the public narrative often tends to focus on the considerable risk of new technologies [20–22]. Public debate can have a tendency to oscillate between unrealistic expectations on one hand, and potentially overblown fears on the other. Overall, there is considerable uncertainty about the short- and long-term implications of algorithms for society, as well as the relationship among the different risks that such algorithms might pose. Often the public debate is dominated by existential fears (such as autonomous weapons systems going haywire), rather than by more immediate concerns regarding privacy infringement and bias (such as large-scale breaches of personal financial data [23], the exploitation of personal information for political purposes [24] and the sharing of personal health data between publicly and privately funded entities [25]).

Below and throughout this discussion meeting issue, we aim to disentangle some of the key aspects of automation and algorithmic decision-making. These include algorithmic accountability and liability; data privacy and security; data governance and consent to use; and transparency and fairness of algorithmically automated decisions. At the same time, a common language across relevant technical areas is needed, in order to understand and make collective progress on these and other challenges that arise as algorithms have an increasing impact on society. Finally, as algorithms become more powerful, and data collection even more widespread, government regulation is beginning to emerge. The European Union's General Data Protection Regulation (GDPR) [26,27], which came into force in May 2018, is a key example. A much-debated aspect of GDPR is the regulation of automated decision-making; that is, algorithmic decisions that do not necessarily require human intervention. The need for new legal and ethical frameworks for such decisions has also been promulgated elsewhere [28–30]. These and other regulatory questions have prompted considerable policy interest and debate in a number of countries [31–34].

We begin in §2 below by describing the evolving technological context which is driving the impact of algorithms in society. We then consider the following topics:

*§3 Legal and ethical frameworks.* These topics are treated by Blacklaws [2], Hildebrandt [3] and Drew [12] in this discussion issue, and in §3 we also provide additional background on the intersection of ethics and law with recent technological developments in data science. Blacklaws [2] discusses the notion of algorithmic transparency and accountability, Hildebrandt [3] highlights the distinction between code and data-driven regulation and finally Drew [12] describes an ethical framework that governments can consider when managing data projects. We additionally highlight issues we view as forthcoming from legal and ethical perspectives.

*§4 Bias, fairness and transparency.* These topics are treated by Oswald [4], Schuemie *et al.* [9] and Quinn [13] in this discussion issue. This area has received considerable attention in the use of algorithmic summaries for legal decisions [35]. Here we explore the usage of algorithmic tools for assessing risk and the legal precedents this sets for administrative law, as discussed by Oswald [4] in this issue as well as others elsewhere [36,37]. Sources of decision-making bias are well understood in more classical settings, such as medical statistics as discussed here by Schuemie *et al.* [9], where reporting bias can inadvertently be encouraged by the need to achieve a fixed statistical significance level for a result to be published. This naturally affects other branches of science as well [38–41], though implications for data science are only now beginning to be fully understood. Finally, questions of algorithmic fairness and bias will play an important role in defining the potential of data science in developing countries, as discussed in this issue by Quinn [13] and by others elsewhere [42].

*§5 Accountability and agency.* These topics are treated by Reed [5] and Shah [6] in this discussion issue. Fairness as described above has a natural foil in unfairness; if an algorithmic decision in unfair then some agent must be accountable (for instance, the institution using an algorithm, as suggested by the Association for Computing Machinary [43]). Reed [5] considers legal liability in this issue, arguing that small steps in legal modifications are less risky than the rapid large-scale development of a new legal code. Shah [6] discusses accountability in detail, and as we note in §5, the surrounding issues are complex [36,44–47].

*§6 Governance of data and privacy.* These topics are treated by Nissim and Wood [7] and Azencott [8] in this discussion issue. Data governance refers not only to the principles that determine how we store and share data while respecting privacy, but also how we use data to make decisions [25,28,48]. Nissim and Wood [7] give a technical discussion of privacy in this issue, which we augment in §6 with a discussion of data theft and unintended losses of privacy. Each of these areas is evolving rapidly as technological constraints change. For example, by combining data sources we can learn more than would be the case from analysing any one source independently [49,50]. Yet data science has yielded analysis techniques by which private information can be inferred from a combined analysis even if this information is not directly present in any one source. This presents considerable challenges in important areas such precision medicine—discussed here by Azencott [8]—since the requirements of aggregating health data across a population and then personalizing medical decisions can be directly at odds with privacy.

*§7 The impact of algorithms on human behaviour.* These topics are treated by Mikhaylov [10] and Bastos [11] in this discussion issue. Privacy as described above is just one avenue where algorithms, social science and human behaviour intersect [15,51]. In §7 we explore the growing impact of algorithms on human behaviour and the societal impacts of algorithms [29,42,52]. In this issue Bastos [11] treats the case of humans interacting with bots on social media and the resulting repercussions for public dialogue. Mikhaylov [10] describes the opportunities that can emerge when data scientists and government entities collaborate.

We conclude in §8 by drawing the above considerations together. We note that the diverse set of topics we have lead the reader through are often bundled up as one single consequence of technology. Many of the more problematic aspects at the interface between technology and society come as the consequence of rapid and significant technological progress. Further debate is needed to arrive at a coherent approach to such widely differing aspects of letting algorithms impact our daily lives.

## 2. Technological innovation and context

It is natural to ask why the current vogue for posing difficult ethical questions regarding the usage of algorithms in society has arisen. The answer is that a number of technological changes have rapidly been taking place, which together have been paving the way for algorithms to impact society. First, we have moved from the setting of data being scarce, expensive and collected according to a pre-specified experimental design, to data being collected incidentally and readily available in large quantities [53]. Data is often not acquired according to any specific experimental design in the latter setting, and so may be biased, and not representative of the entire population under study [54–56]. Nevertheless such data has proven extremely useful, in that it enables us to make at least partial sense of complex generating mechanisms in the world around us.

Algorithms themselves are of course not new. An algorithm is nothing more than a set of instructions to be followed exactly—a simple example being a recipe in a cookbook—and the word itself derives from the name of the ninth-century mathematician al'Khwarizmi. Some algorithms are entirely deterministic, with steps executed the same way when solving any given problem (such as sorting a set of numbers), and others are stochastic, involving some element of randomness or uncertainty (such as shuffling a deck of cards). Numerical algorithms are usually understood in terms of their key properties, such as how much computation and memory they require as the amount of input data scales up, and what theoretical guarantees they can provide on their outputs.

Algorithms are usually constructed with one of two frameworks in mind [57]: either the explanatory framework of writing down sufficiently simple rules (a model) for how the input data were generated, but allowing for random perturbations away from that rule through some stochastic law; or, in a purely predictive framework where the stochastic law is not specified, but we instead aim to ensure that our predictive error for future observations remains low. Lehmann [58] discusses how one can arrive at the fairly complex types of model we often see in the modern sciences, say by combining simpler building blocks. A statistical model is the mathematical recipe for how new observations are generated, usually involving a formula made from predictor variables.

In this discussion issue, Schmueli *et al.* [59] make an even stronger distinction between (causal) explanatory models and those models geared purely towards prediction. Hand [60] highlights the many potential problems with data driven predictions, including the risk of models that are too expressive or flexible, and unpredictable data-driven shocks. An interesting complementary discussion is provided in [61,62], as well as by [63] which considers algorithms less tractable to analysis. In the predictive framework, as long as we can predict the future well, we do not care if our model is simple or informative. There are, however, mathematical reasons why we would assume that a simple model would yield good predictions [64] (see also [65]). The predictive framework is very powerful. It has seen considerable success in scenarios such as web search and product recommendations, and lies behind the rise of predictive algorithms for machine learning. By contrast, the explanatory framework often proposes an explicit structure in the data, such as a straight line dependent on some explanatory variable perturbed by some unexplained variation in the observations. Future values of the data can then be explained by the future values of explanatory variables.

Any method for generating predictions can in theory be made arbitrarily complex. As this happens, however, more data is needed to determine its precise form. This used to be a bottleneck which prevented data analysts from using complex models and algorithms. An example is given by neural networks, which build models of how responses depend on variables of interest using input data in relatively complex ways [53,66]. In the past, a lack of computational power and large data sets prevented methods such as these from working well and being used at large scales.

The predictive framework is normally used to solve certain archetypical problems, such as supervised learning (classification, determining the type or class of an input datum), and unsupervised learning (clustering, or detecting groupings or patterns in input data). In the former setting, algorithms learn from known, grouped examples to refine their classification of a new data point. In the latter setting, algorithms aim to classify data points without knowing what any of the groups are a priori.

In the mid 2000s, algorithms that had not previously been implementable at scale became both feasible and effective, because of faster computers with more processing power and also, crucially, more data [53,67]. In particular, neural networks became competitive, solving problems in speech recognition [68], image recognition [69] and language translation [70]. These algorithms solve well-defined tasks, but they do not necessarily provide an interpretable representation of what they are doing. The methods are often referred to as “model-free” (also called “non-parametric” or “black box methodology” [20]) methods. Strictly speaking, often there is a very flexible notion of a model hidden in the conceptual design of these methods. The methods allow us to approximate the system we are studying, without being able to write down a simple generating mechanism. Classical understanding of approximations is linear—e.g., using the data once to form a prediction as a superposition of basic building blocks. By contrast, a common mechanism in neural networks is to iterate, using the input data repeatedly. This makes a mathematical understanding of such methods much more complicated.

The contrasts between predictive and explanatory frameworks brings a natural tension. The scientific method favours simple expressions where relationships between multiple variables can be simply understood and quantified. Yet, if all that is needed is to predict future values accurately, then this is not a necessity. Trouble arises when these goals conflict, and there are hidden relationships between variables that may obscure the rationale for how predictions are being made. For example, imagine an algorithm that makes hiring decisions on the basis of CVs. Now suppose we allow the algorithm to consider the font of the CV as an input. How comfortable would we feel basing a hiring decision on the font used in a CV? This may have explanatory power, but somehow to our minds it should not contain relevant information for the purposes of hiring. The example of using a seemingly information-free variable like the font in a CV also brings up the problematic nature of non-transparency. What if there were a correlation between gender or ethnicity and the choice of font? If we do not understand what relationship is being hypothesised as part of an algorithmic decision, then it becomes nearly impossible to determine when an algorithm is capturing structure that is spurious or should not be considered.

Traditionally humans have focused on understanding simple relationships between few variables. This is somewhat of a fallacy: as we have looked for them, we have discovered natural laws that seem to represent simple relationships between observed quantities, seeming to indicate that such simple relationships are fundamentally important. Yet if the true relationships are very complex–for example, our planetary weather system—could we ever in a data-poor setting discover them? Historically science has often appealed to the principle of Occam's razor, which suggests that if several equally valid explanations are on offer, then the simplest should be chosen. Yet what if simple explanations offer no ability to predict? It is not clear if machines should be built to resemble humans [71], looking for simple overarching frameworks. In fact a basic usage of computing is to keep track of many more very complex structures than we are able to as humans.

Without the simplicity of Occam's razor encouraging us to see a simple relationship between a few variables, it is also hard to understand what to do with unusual observations. Often in large populations it is easier to find outliers lying far from the main body of observations, and involving ranges of predictors that no other observations involve. Very little can be guaranteed about predictive or statistical performance when this is true. As models become more more complex this effect is exacerbated further. In such settings, one can imagine scenarios in which decisions ought to be made instead on what could be considered ‘fair’ treatment, based on offering the options available to the nearest part of the bulk of the population.

Furthermore, there is a danger when algorithms offer only mechanisms for predicting future values, rather than any means of giving understanding as to why a certain value is predicted. Most often we can only observe phenomena, without the ability to control the experiments we are studying. Establishing cause and effect is much harder [72]. Yet, to control the systems and environments we live in, this determination is often necessary. An apparent association between variables does not imply that changing the level of one causes the level of another to change. This is because observing two variables that are changing is different from observing the effect of their changes after one of them has been caused to change. A number of logical fallacies follow unless care is applied to such problems. Spurious correlations between variables can easily appear in practice, making it especially difficult to make strong deductions from data. The area of causation has been the focus of considerable recent attention, especially in machine learning [72].

## 3. Legal and ethical frameworks for data management and algorithmic decision-making

The appropriate ethical frameworks for both data governance and algorithmic governance are discussed in [28], and overarching principles are provided by [43] as well as [73]. The overarching principle recognised by the British Academy and Royal Society report is striving towards human flourishing; e.g., the purpose of exploiting data, and establishing algorithmic exploitation is overall solely for human benefit. Such an overarching ethical principle is also adopted by [29] and [30]. Despite this seeming perhaps straightforward and simple, a number of thoughtprovoking questions arise at a more granular level. Legislation does exist for subsets of this setting, even if their interpretation still needs to be fully fleshed out.

In May 2018 the new General Data Protection Regulation (GDPR) became law throughout the European Union. This regulation has a number of facets, and details can be found at the ICO https://ico.org.uk/media/for-organisations/documents/2013559/big-data-ai-ml-and-data-protection.pdf. There is a new concept of a *lawful basis for processing*, as well as guidelines on automated individual decision-making and profiling. Additional explanation of the application of machine learning is directly available from the ICO https://ico.org.uk/media/for-organisations/documents/2013559/big-data-ai-ml-and-data-protection.pdf.

When decisions are made automatically, the data processor must ensure (that if requested) human intervention is available, as well as an explanation. GDPR recital 71 in fact states: ‘The data subject should have the right not to be subject to a decision, which may include a measure, evaluating personal aspects relating to him or her which is based solely on automated processing and which produces legal effects concerning him or her or similarly significantly affects him or her.’ This seems like an overall exemption from automated processing, but the regulation also specifies which scenarios are in turn exempt from this specification (for example, when subjects given their assent). Much has been written about the ‘right to an explanation’, language which by some authors is considered to be somewhat imprecise [74].

GDPR is also discussed by Blacklaws [2] in this discussion issue. In particular, the predictability and clarity of opaque complex algorithms can work against easily generated explanations. An open question is if the predictive framework can produce an explanation—an area which is the subject of considerable research [49,75], as is the interpretation of the right to an explanation for more opaque systems [74,76].

In contrast Hildebrandt [3] here discusses deterministic legal rules distilled from law, versus using legal texts as natural language processing data. This brings up a different overlap between technology and society. The distillation of law into a machine learning or deterministic system would partially remove the need for human interaction when dispensing the law (see also [77]). A whole host of new problems arise due to displacement, especially relating to accountability, as we discuss in a later section.

Drew [12] in turn discusses ethics in terms of design, highlighting the need to demonstrate public value. We also note the recent development of ethics boards inside many of the companies whose core businesses rely heavily on data [78]. We imagine there are scenarios in which connecting best practice of those ethics boards to an independent body might help to ensure fair and equitable practice.

## 4. Bias, fairness and transparency

Bias in automated decisions is a key concern [79]. There is an implicit assumption that once we collect enough data, bias will no longer be a problem—an assumption that in general is not justified. Bias can arise in algorithms for at least two reasons: first, the data we have collected may have been preferentially sampled, and therefore the data sample itself is biased. This has arisen for example in a situation highlighted by [54], where the use of mobile phones to report problems with city streets led to a spatially biased sampling reflecting the density of phones rather than of street problems. Similar examples abound in other settings [55,56,80,81].

Bias can also arise because the collected data is reflecting existing societal bias [82]. For example, rates of arrest may differ by race [37]. Very careful analyses are required in the scenario when the rates of certain events depend on a group affiliation [36,37,83]. This problem has flavoured many debates about automated legal decision-making.

Oswald [4] discusses in this issue the use of risk assessment tools as decision aids, and specifically the application of random forest algorithms. Random forests can be very opaque in their description of a process, as ‘model-free’ tools that can approximate the generating processes of input data. Oswald describes ‘procedural’ fairness, namely that the procedure by which a decision is arrived at is consistent and does not vary across cases (this can be thought of as an operationalization of fairness [84]). Of course, there are many other ways in which notions of fairness can be described. Others [37,85] consider the notion that the probability of a certain event should not depend on information that society has deemed irrelevant—such as race or gender. In some settings such information can appear to have predictive power, but at the same time legal strictures may preclude its use. Other examples include causal methods of defining fairness [86], in particular introducing the concept of replicating an individual with different characteristics and seeing if decisions alter.

Even with causal methods, the data available in any particular scenario may not contain the information required to make a decision fair. This can mean for instance that de-biasing is not possible [87]. The lack of uniformity of data can also mean that it is not possible to ensure an equal degree of privacy to all individuals in a data set [84]. Fairness can be considered both for individuals or for groups. For example, Dwork *et al.* [83] argue that similar individuals should be accorded similar treatment. Fairness can also be defined for groups, where group membership should not determine the type of treatment [88]; however, group fairness often fails [37]. There may also be trade-offs between privacy and fairness: if some attributes are removed from analysis, it is possible that no algorithm can ensure fairness if the data have correlations between protected attributes and the information that has been removed from the analysis. Without having this sensitive information available to us, then no correction for bias is possible [84].

Transparency of algorithmic decision-making has also arisen from a legal rather than purely a technical standpoint [4,89]. Oswald [4] discusses that an explanation has to be ‘adequate’. By ‘adequate’ an explanation has to show why a decision was arrived at. Our standards here, it can be argued, must reflect the context of the decision. If a decision is of minor importance, we may chose to brush its explanation aside in favour of a very good average performance. However, if someone is denied a mortgage or parole, for example, a greater degree of transparency is most likely required. In other sectors we are satisfied to trust the workings of technology without understanding it fully—combustion engines, drug efficacy—but even in such settings we expect there to be standards of safety and of reasonable performance. Public opinion is still being formed; one could for example look at the range of possible performances in the population of interest (say, the effects of the worst 10% of erroneous decisions versus the best 10% of correct decisions). In future it will become important to decide upon the adequacies of deterministic algorithmic guarantees versus controlling simply the statistical performance of algorithms in aggregate.

Unfairness can in these types of problems also arise because not everyone has technology available to them. Quinn [13] discusses the usage of algorithms for improving humanitarian relief using open data sources such as remote sensing imaging. The general push for open data has the potential to eliminate unfairness in this setting. Examples of unfairness is one where data density varies with wealth, such as the aforementioned example of identifying the need for street repairs [54]. The public debate includes examples with disparate impacts in low- and middleincome countries [90,91], and clearly our understanding of global data flows and their impacts is still in its infancy.

Finally, fairness is intricately related to the notion of reproducible research in science. If scientific results are not reproducible, the question of fairness becomes moot, as everyone is in some sense potentially treated arbitrarily. Discussion has been ongoing in the social sciences in this area [38–40]. In part, this is a consequence of the statistical analysis tools for observational data not having caught up with the large volumes of such data that are now routinely collected, as well as publication biases. Schuemie *et al.* [9] explore, using a version of meta-analysis, finding the appropriate way of combining multiple observational studies, and coping with the fact that the empirical distribution of *p*-values is nothing like the analytic null models we are used to applying. Much is needed in the way of new mathematical theories and statistical methodologies to handle scenarios such as these, and it seems that many lessons can be gleaned from considering the challenges faced by observational studies in human health and in the social sciences.

## 5. Accountability and agency in algorithmic decision-making

Accountability of algorithmic decision-making is a popular topic of investigation. A dictionary definition of ‘accountability’ will tell you that the word corresponds to the state of being liable or answerable. The importance of accountability is highlighted in several recent reports [43,73,92]. The Public Policy Council of the Association for Computing Machinery [43] notes the importance that institutions that use algorithmic decision-making are responsible for the decisions they make. The European Union's GDPR outlines rights relating to provisions on solely automated decisions, as well as profiling. Article 22 of that regulation describes the rights of individuals in the setting of automated decisions. For example, there are scenarios where such processing is permitted; e.g., upon entering a contract, with the individual's consent, or in ways stipulated by a member state.

The rights of the individual include being able to challenge automated decisions: individuals should be able to obtain information on the processing of their data, but there should also be mechanisms for human intervention (the ’human in the loop’ discussed also by [93]), and individuals should be able to challenge algorithmic decisions. This is potentially a very difficult area, as algorithms may be very data–driven, opaque and complex. It is hard to enforce accountability without transparency. Yet simply listing the steps of an algorithm may not be sufficient (e.g., ‘A ten layer neural network was trained, where performance was assessed in terms of mean-squared error.’). Such an explanation offers the semblance of transparency, but very little meaning. And even to an expert, without access to the data used to determine the algorithm's outputs, it would be hard to decipher what had happened [75]. A natural pressure point is thus the complexity of modern algorithms, which may mean that de facto it is very hard to challenge algorithmic processing techniques as well as the decisions to which they give rise. This is not an easy topic, as more complex techniques often yield better performance but are harder to untangle and adequately describe.

Linked to accountability is the concept of agency. By ensuring citizens’ agency in the usage of automated processing, one attempts to give control back to individuals. One would expect to see standards of an acceptable degree of transparency and accountability developed for different public and private sectors. In some sectors, measures of statistical performance may be acceptable. Standards will then also be necessary for pre-processing and cleaning data—these steps are very nearly always necessary to make any algorithm work, but might be likely to unfairly influence decisions for segments of the population that lie outside the mainstream.

In this issue, Shah [6] advocates standards in checking for bias and cautions against the universal panacea of apparent transparency, instead proposing to look for differential impact. Shah suggests ensuring societal mechanisms so that algorithmic decisions can be challenged. Linked to challenging algorithmic decisions is the notion of liability, either civil or criminal. Reed [5] advocates using liability as the first approach to regulating algorithmic decision-making. Reed proposes ex post analysis of how a decision was arrived at, and suggests this may be sufficient to determine how any person treated wrongfully can be compensated, even though predicting this outcome might have been very hard. A challenge with this approach is that disentangling the motivation of a particular decision ex post can be difficult. As most decisions are based on large volumes of data, it may be hard to find the true motivation or deciding factor in an algorithmic decision; when large masses of data are present, many attributes in the data may behave similarly and so can be hard to separate out. Technical standards may need to be developed to guide how such an analysis might be implemented in a manner to avoid confounding between effects.

Finally liability may be difficult to determine. To illustrate this, let us discuss the differences in the role of algorithms (the recipe for a dish) relative to the role of data (the necessary ingredients, which could be of good or poor quality). The dish may taste poor because the ingredients were not of good quality, or because the recipe was poor. In the same way, automated analysis is naturally vulnerable to poor quality input data. One example might be a lack of understanding of how the phenomenon of interest changes over time—the limitations of Google Flu trends being a case in point [51]. Also if the data analyst is not the data controller, then additional problems can arise. What happens if the supply of the data may be susceptible to disruption? With less data, performance of an algorithm can be worse, but at what point does one set a criterion to determine if a decision should be voided or not based on acceptable algorithmic performance versus speed of service. Moreover, algorithm design involves a number of choices–most crucially that of the metric for ‘performing well’. This could be average performance (most users will have a good outcome), allowing for robustness (the worst outcomes will be limited, with some sacrifice of average performance) or even taking computational feasibility and analysis time into account in the metric. In general it is not feasible to design an algorithm which is optimal with respect to all metrics at once, and so which design choices might make an algorithm designer liable for negligence?

## 6. Governance of data and privacy

Algorithmic decision-making is concerning in part because of worries about rapid and automated decisions being made without the benefit of human judgment. Equally concerning from a privacy perspective is the proliferation of data; for example, it can be possible to infer highly sensitive and private information about individuals on the basis of social media data [50].

In this issue, Azencott [8] discusses the benefits in cases where data need to be shared for health innovation across a population, but where there are still concerns about privacy impacts on individuals as a consequence of the data being shared. A new facet is that during the life expectancy of an individual born today, we might expect data to remain available (and perhaps relevant) for the next 80 years! New concepts have been introduced such as time-varying consent for data usage [94], where theoretically data could be pulled in and out of studies at any time.

In contrast, Nissim & Wood [7] discuss the meaning of privacy, and how technical definitions of the concept may not necessarily match non-technical desiderata. Understanding which degrees of digital privacy are possible is fundamentally a technological question, and yet it must be joined by considerations of specific notions of privacy that can differ by culture.

Other contemporary work focuses on high-level considerations, such as the notion that all data use should be guided by the principle of ‘human flourishing’ [28]. The report of [28] recognises that recent technological developments have strained existing regulation. Clearly recent events such as data use for political purposes [24], data breaches such as [95] and discussions regarding the use of public data in public-private partnerships [25,96] all highlight this problem. A key theme common to these three examples is the ‘at scale’ aspect: the UK National Health Service shared data of 1.6 million patients, data sharing between Facebook and Cambridge Analytica is alleged to have involved over 80 million users, and a recent credit ratings agency data breach at Equifax concerned nearly 150 million customers. What is also thought-provoking in these examples is the richness of information; in the alleged Facebook scenario, potentially previous website visits, information about friends and various personally identifiable information about individuals and their friends could have been shared. Another natural concern is the spread of the information that is not directly observed, but is deducible from what has already been shared [32,50,97], especially if multiple data breaches concern the same individual.

What can then be done? First of all, the three examples cited above did each elicit considerable societal response. The Information Commissioner's Office in the UK has become involved as well as other agencies; discussions have similarly begun regarding UK National Health Service data that has been shared; and finally much has been written about the challenges facing Equifax and other commercial entities in light of data breaches [23].

Second, the question of jurisdiction will become inevitable, especially with the scale of international breaches. Equifax and Facebook are internationally functioning bodies, and so impact citizens internationally. The European Union may have been one of the first entities to implement modern data protection regulation, but inevitably most questions and legal concerns about data usage will strongly involve notions of national boundaries.

Finally, various bodies and professional societies have proposed standards to guide data governance and privacy. For example, the British Standards Institute has already proposed a standard for the ethical design of robotic devices (BSI BS 8611:2016 Robots and robotic devices); there are also multiple IEEE standards (https://ethicsinaction.ieee.org/) projects in this area. Should regulation lag behind or prove difficult, establishing standards may be a soft approach to the topics of data governance and privacy. In fact, this type of approach may well mirror suggestions offered by Reed [5] in this issue.

## 7. The impact of algorithms on human behaviour

Human behaviour is often the subject of data collection. Savings in terms of costs and general utility will no doubt accrue after further application of analytics to this type of data. Concerns in terms of using such data are often in terms of the monopoly of a few entities in being able to collect such at-scale observations [14]. Entities in the commercial sector are increasingly instituting their own ethics committees [78] to allay public concerns. The notion of a monopoly is now being discussed in terms of data, as it becomes increasingly clear that the diversity of human behaviour requires large volumes of data to be understood and reproduced faithfully in algorithmic contexts. The hope of understanding ever-increasing complexities of human behaviour [98] has driven this set of developments.

In this issue, Mikhaylov [10] discusses policy delivery using data science and artificial intelligence, and its impact on public services (see also [33,99], the latter of which considers barriers to automation via artificial intelligence). Impacts, including most prominently the notion of increased automation, have raised concerns of large-scale job losses, and numerous other reports have been forthcoming, given the societal importance of this possibility [77,100–103]. Different scenarios are described in [102] depending on relative hardware and software costs, labour market dynamics, and also societal acceptance of automation.

Automation and algorithms in society are strongly intertwined. In popular images of automation, the automatic solution is normally a robot—a corporeal manifestation—while in many practical scenarios such as customer support, an algorithmic solution will be equally (or even more) viable. The physical ability of the robot will depend on the analytics of its algorithm, which will impact its ability to function well. Automation stands potentially to impact many white collar jobs [77] such as accountancy and the legal profession. It seems likely that such algorithms will interface with human decision-making, a development necessary to gain societal acceptance and thus wide-scale use. The societal impact of such developments is of course notoriously hard to forecast.

Bastos [11], also in this issue, discusses other aspects of the human–algorithmic interface, some of which have also been described in the analysis of political polarisation [104,105]. Bastos explores how sentiments are spread and amplified by sockpuppets. Further problems would involve how to detect when algorithms are trying to modify human behaviour [24,106]. Many interesting future questions arise from algorithms masquerading as humans [107], requiring new technological (algorithmic) solutions to be developed. Presumably, developing algorithms to determine when non-human behaviour is present can help to detect whether if one is interacting with a human or a machine. Indeed, it is easy to imagine a future in which societies might propose laws requiring that algorithms identify themselves as non-human actors at the start of any interaction.

## 8. Discussion

This discussion issue has highlighted a number of the questions that arise as a consequence of automation and algorithmic decision-making in society. In particular, the issue has considered implications of the usage of algorithms in terms of legal and ethical frameworks [2,3,12], bias and fairness [4,9,13], accountability [5,6], governance of data and privacy [7,8] as well as algorithms that interface with, as well as reinforce, human behaviour [10,11].

As the development of technology outpaces the development of ethical and regulatory frameworks, the consequences of technological advances are rapidly becoming apparent. In many instances we have not yet developed the regulatory structure or best practice to keep pace with technological innovation. This lack of balance in development causes back-and-forth seesaw cycles in the broader debate on the role of algorithms in society, interspersing an enthusiasm for technological progress with strong concerns for privacy and ethics. The influence of this dual set of priorities (technological and ethical) is discussed in detail throughout this discussion issue (see, e.g., [5,8]). Informed interdisciplinary debate should help provide pathways to mitigate the seesaw, by outlining the trade-offs in a clear and considered fashion.

The law interfaces with the development of algorithms on several levels. First, certain aspects of the law can be automated and operationalised by algorithmic developments, as discussed by [3], who asks whether the machine learned principles from data could be counted as regulation. Blacklaws [2] discusses both accountability and transparency in the context of algorithms. Shah [6] in turn emphasises the need to achieve public trust in order to operate.

Reed [5] asks fascinating questions about the efficacy of preemptive legislation and instead highlights the role of personal liability in this setting. As for interfacing with administrative law, Oswald [4] probes the question of fair practices in such a context, and what might be considered fair. Drew [12] in turn advocates for ethical design drawing upon previous practical experience.

These questions of governance of data and algorithms are especially challenging given the current at-scale implementation of many algorithms in the commercial sector, and the longevity of data that can be stored and re-used. Here the importance of an individual's consent to data usage must be balanced to some degree by societal norms and delegated authorities. It may not be feasible that every active individual in society is at every moment involved in negotiating with other parties the terms of how their data may or may not be used.

In fact, many of the ethical issues debated in this discussion issue touch upon the availability of large but messy volumes of linked observational data. Large volumes of data have been collected, often without a rigorous collection design, and the general public is sharing information about themselves, often without a clear understanding of the risks and opportunities involved. With new legislation and heightened awareness, the proliferation of data may be abating, but already considerable amounts of information are available about each of us. In this context, Nissim & Wood [7] ask the question of what the nature of privacy is precisely. The repercussions of the availability of large and detailed personally identifiable information, is that much more can be inferred from them than seems to be available at first glance.

However, the value and cost-savings in using data lies exactly in being able to combine it both across data types, and many, many individuals. If given segments of the population refrain from making their data available, then we cannot make sense of the population as a whole, or understand its variability. Azencott [8] asks how to balance privacy concerns with the need for large and detailed data sets to answer questions about health, and the growing area of precision medicine, which requires both detailed and linked personal data. In the context of human health, societal interest has moved beyond privacy to question the trustworthiness and reliability of analytical algorithmic tools—a question that can be asked and discussed at scale, as do Schuemie *et al.* [9] here.

Algorithms operating at scale also have an impact on policy, political and social discourse. Mikhaylov [10] highlights the promise of analytics in the public sector. Quinn [13] shows the policy utility of machine learning for image processing in refugee camp mapping. Finally Bastos [11] focuses on understanding the impact of social media on recent elections. Bastos specifically considers bot detection, and characterising botnets in the context of the UK's Brexit referendum campaigns.

What then is clear from the discussion and sets of examples cited above? Fundamentally, the interface of policy and algorithmic development is still evolving. Initial attention has focused on a set of distinct issues that have gained popular interest: algorithmic bias, privacy and the economic dangers of automation more generally. As technology develops, new interfaces will be found. Continued debate is needed to guide the development not only of technology, but also of the policies that enable its use. We envision that this discussion issue will generate further debate on the current set of critically important topics as well as stimulate key questions that have yet to arise in this area.

## Data accessibility

This article has no additional data.
